# Smoking prevalence and correlates among inpatients with schizophrenia or schizoaffective disorder

**DOI:** 10.1038/s41598-025-93256-2

**Published:** 2025-05-27

**Authors:** Caili Wu, Paul Dagg, Carmen Molgat, Nataliya Grishin

**Affiliations:** 1 Tertiary Mental Health & Substance Use Services, Interior Health Authority, Kamloops, 909 3rd Ave, BC V2C 6W5 Canada; 2 Department of Psychiatry, University of British Columbia, Vancouver, BC V6T 1Z4 Canada

**Keywords:** Cigarette smoking, Schizophrenia, Symptoms, Cognitive function, Comorbid substance use disorder, Tertiary mental health care, Psychology, Human behaviour

## Abstract

Many studies have shown that cigarette smoking prevalence rate is high in patients with schizophrenia. Despite the strong association between smoking and schizophrenia, findings on the relationships between smoking, psychiatric symptoms and cognitive functions remain mixed. Furthermore, the smoking rate among acute inpatients who need tertiary mental health care is still unknown. In this study we investigated the smoking rate in this patient population and examined connections between smoking and cognitive functions, psychiatric symptoms, and clinical and demographic characteristics. A retrospective chart review of patients admitted to a tertiary acute psychiatric facility over a 7-year period was conducted. Information such as patient smoking status, diagnosis, and psychiatric assessment scores, were retrieved. Independent samples t-tests and Chi-squared tests were used to compare variables between smoker and non-smoker groups. The smoking prevalence rate was 72%, approximately four times the smoking rate in the general population in Canada. Compared to the non-smoker group, the smoker group were significantly younger, more likely to be male, had less years of education, shorter illness duration, higher rate of concurrent substance use disorder, and less days of hospital stay. However, the two groups did not show differences in severity of illness, types/numbers of medication used, positive and negative symptoms, and cognitive impairment. Smoking status appeared to be associated with several demographic and clinical features. Smoking did not significantly relate to patients’ illness severity, medication use, psychiatric symptoms, or cognitive functioning.

## Introduction

Numerous studies acrossdifferent clinical settings have reported that cigarette smoking prevalence is markedly high in patients with schizophrenia and schizoaffective disorder. About 65–90% of patients with schizophrenia smoke regularly^1–6^. Smoking prevalence is not just higher than in the general population, but also higher than in patients with other psychiatric disorders such as bipolar disorder and major depressive disorder^7–10^. Smokers with schizophrenia also have more difficulty quitting smoking, are more likely to be heavier smokers than smokers without mental illness. Onset of regular smoking often precedes the onset of schizophrenia^8,11–16^. Smoking seems to be consistently associated with younger age and lower education^2,3,17,18^. Smoking is significantly associated with co-occurring or history of substance abuse^2,8^.

The concurrence of schizophrenia and smoking has inspired research into linkages between smoking and clinical symptoms, cognitive functions, psychiatric comorbidities, as well as demographic characteristics. However, studies investigating the relationship between smoking and psychiatric symptoms have found mixed results. One line of research demonstrates that smokers have more severe global psychiatric, negative, and/or positive symptoms than nonsmokers^5,18–22^. Other studies show decreased negative symptoms among smokers, i.e., nonsmokers have more severe symptoms^17,23^. Yet another line of research found no difference between smokers and non-smokers regarding positive and/or negative symptoms^2,18,20,23–26^. Nevertheless, there seems to be a connection between smoking and depressive symptoms^3,20,27^. Additionally, there is robust evidence that smoking may be one of the major risk factors for the reduced life expectancy in people with schizophrenia, emphasizing the need to better understand the dimensions of smoking in people with schizophrenia, and targeting interventions appropriately^1,2,4,16^.

Existing findings on the relationship between smoking and cognitive functions in patients with schizophrenia are also inconsistent. Some studies report that smoking has positive effects on cognitive functions such as improved divided attention, processing speed, working memory and executive function^7,25,26,28,29^. On the contrary, some studies find smokers have more cognitive impairment than non-smokers, for example, worse performance on problem solving and lower visual sensitivity^3,6,18,19,30^. Still other studies show that smoking has no significant impact on cognitive functions^23,29,31,32^. It is noteworthy that a wide range of cognitive tasks were selected across those studies, which might be a contributing factor to the mixed results.

Several hypotheses have been proposed to explain the high prevalence of smoking in schizophrenia and other serious mental illnesses. However, no single hypothesis can provide the full interpretation and hypotheses are not mutually exclusive^4,29^. The most widely accepted theory suggests that smoking is a form of self-medication for reducing psychiatric and cognitive symptoms, as well as side effects of antipsychotic medications^28,33–37^. There has been considerable supportive evidence of the self-medication hypothesis but it is also challenged by the results of many other studies^6,29,31^. Another popular theory is the shared vulnerability hypothesis, which assumes that shared genetic or environmental factors and neurological deficits inherent to the pathophysiology of schizophrenia make people with schizophrenia more vulnerable to tobacco use^38–40^.

In summary, the evidence suggests that smoking prevalence is consistently high in patients with schizophrenia. However, findings on the relationships between smoking status and demographic characteristics, cognitive function and clinical symptoms are complex and mixed. Many related studies have been conducted in various clinical/community settings, including both inpatients and outpatients. Yet research investigating the association between smoking status and cognitive functions, clinical features, and demographic characteristics within a single sample is limited. Most studies have focused on one or two aspects of the relationship between smoking status and cognitive functions/ psychiatric symptoms.

To date, no published research has investigated the prevalence rate of smoking among acute inpatients who need tertiary mental health [TMH] care. As psychiatric deinstitutionalization moves forward, largescale long-term psychiatric hospitals have been replaced with comprehensive community-based service continuum. In the multi-level pyramid framework model of mental health service organization proposed by the World Health Organization, TMH services was positioned at the top of the pyramid, while the bottom level being self-care^41^. Tertiary care is defined as specialized services delivered by highly trained multiple disciplinary professionals to individuals with illness that are complex and refractory to primary and secondary care^42^. In other words, patients were referred to TMH services when they can no longer be treated successfully in hospitals or community cares. Their psychotic symptoms are usually more complex and severe. Investigating the smoking status in a patient group with acute, severe, complex, and treatment-refractory mental illness is indeed a critical and challenging task. This population often has unique needs, and understanding their smoking behavior could provide valuable insights into their treatment and overall care.

Taken together, the current study aimed to (1) examine the prevalence rates of smoking among acute inpatients with schizophrenia in TMH care, and (2) further investigate the relationship between smoking status and psychiatric symptoms, cognitive functions, demographic characteristics, and several clinical measurements such as illness duration and number of antipsychotic medication used.

## Methods

### Clinical setting

The study was conducted at the acute tertiary mental health facility, located in Kamloops, British Columbia, Canada. This facility provides highly specialized mental health services for individuals with complex and severe mental illnesses, and who cannot be cared for in the secondary or general hospital psychiatric system. The facility offers three specialized programs: Adult, Geriatric, and Neuropsychiatry. Admission is exclusively by referral, with patients being referred from the six general hospitals across the Interior Health and Northern Health Authorities of British Columbia. The current study only involved patients admitted to the Adult program. The admission criteria included but not limited to: Primary diagnosis was psychotic disorder or affective disorder; higher degree of disability; unable to be managed in the community with current resources; and inpatients of psychiatric units and psychiatric assessment units of general hospital. The facility has four on-site psychiatrists, whom are FRCPC (Fellow of the Royal College of Physicians of Canada) designated.

### Participants and measures

This study was approved by the Interior Health Research Ethics Board and the University of British Columbia Clinical Research Ethics Board. All methods were performed in accordance with the requirements of the Tri-Council Policy Statement: Ethical Conduct for Research Involving Humans (TCPS2, 2014).

A retrospective chart review of medical records for patients admitted to the adult program from October 1, 2011 to September 30, 2018, was conducted. For each patient, the psychiatrist performed a detailed assessment that included demographic information such as age, clinical features such as diagnosis, and several psychiatric assessment scores at admission. This information was subsequently entered into their electronic medical records. If a patient was re-admitted to the facility during the study period, only the first entry record was included in the data analysis. The chart review was performed in two steps. Firstly, patient smoking status and diagnosis information was identified to calculate the smoking prevalence rate in different diagnostic groups. The diagnoses were made by the onsite psychiatrists according to the Diagnostic and Statistical Manual of Mental Disorders, 4th Edition (DSM-IV). Second, for patients diagnosed with schizophrenia or schizoaffective disorder, their medical records were further reviewed for demographic information (i.e., age, gender, and education in years), clinical characteristics (i.e., illness duration, number of psychotropic medication used, comorbid substance use disorder (SUD) status), and length of stay (in days) at the facility. Lastly, scores of several psychiatry assessments were retrieved. The assessments include the Clinical Global Impression scale (CGI), the Positive and Negative Symptom Scale (PANSS), and the Montreal Cognitive Assessment (MoCA). The CGI scale evaluates the severity of illness. The MoCA measures the overall cognitive impairment in patients with schizophrenia. The PANSS assesses general, positive, and negative symptoms.

The facility maintains a smoke-free environment. All patients were assessed using standardized nursing assessment packages, which include screening for nicotine use. Inpatients who use tobacco are identified at admission and offered support, such as nicotine replacement therapy (NRT), to help manage withdrawal symptoms. Patients who smoked and chose not to participate in NRT were allowed to smoke during their passes in designated outdoor smoking areas.

### Statistical analysis

Data was analyzed using IBM SPSS Statistics 25. Descriptive statistics of patient smoking status and demographic information, as well as cognitive and clinical characteristics assessed by the CGI, MoCA, and PANSS were generated. Independent samples t-tests were used to compare the assessment scores, age, and years of education of the smoker and non-smoker groups. Chi-squared tests were conducted to compare category variables such as gender distribution of the two groups.

## Results

### Smoking prevalence rates in patients with schizophrenia and other psychiatric diagnoses

After removing duplicated records, smoking status information was available for 391 unique patients. According to the DSM-IV criteria, 60% (233) of the patients had a primary diagnosis of schizophrenia or schizoaffective disorder. Of the remaining primary diagnoses, 11% (43) were bipolar, 6% (25) were major depressive disorder (MDD), and 23% (90) were other psychiatric diagnoses.

For patients with schizophrenia or schizoaffective disorder, 66 (28%) were non-smokers while 167 (72%) were smokers. Therefore, the smoking prevalence rate was about 72%. We also calculated the smoking rates in other diagnostic groups: the smoking rate was 67% for bipolar disorder, 40% for MDD, and 58% for other psychiatric disorders. A Pearson chi-square test indicated that the prevalence rates differed significantly between the groups, χ^2^ (4, *N* = 391) = 30.368, *p* < .000.

### Smoking status and demographic characteristics in patients with schizophrenia

For patients with schizophrenia or schizoaffective disorder, two groups (smoker vs. non-smoker) were classified by whether the patients were current smokers at admission. The relevant descriptive and group comparison statistics are summarized in Table 1, “Smoker” and “Non-smoker” columns.

**Table 1:** Demographic, clinical, and cognitive characteristics, and the comparison of those variables between smoker and non-smoker groups.

Variable	Schizophrenia (*n* = 233)	Smoker (*n* = 167)	Non-smoker (*n* = 66)	t/ χ^2^ value	*p* value
	Mean (SD)	Mean (SD)	Mean (SD)		
Age (years)	36.97 (12.57)	34.82 (11.33)	42.42 (13.94)	t(231) = 4.31**	*p* < .01
Education (years)	11.16 (1.90)	10.83 (1.83)	11.98 (1.87)	t(231) = 1.96 *	*p* < .05
Male / Female N (%)	147 (63%) / 86 (37%)	113(77%) / 54 (63%)	34 (23%) / 32 (37%)	χ^2^(1, *N* = 233) = 5.30 *	*p* < .05
Length of stay (days)	55.99 (37.71)	52.96 (31.76)	63.65 (49.16)	t(231) = 4.31**	*p* < .01
Duration of illness (years)	12.53 (10.11)	10.61 (8.31)	17.12 (12.46)	t(127) = 3.48 **	*p* < .01
Illness onset age	23.93 (9.09)	22.98 (8.52)	26.19 (10.09)	t(127) = 1.85	*p* = .07
CGI at admission	4.94 (1.00)	4.92 (0.95)	4.98 (1.12)	t(231) = 0.43	*P* > .05
SUD comorbidity (%)	128 (55%)	107(64%)	11 (17%)	χ^2^(1, *N* = 233) = 42.53 **	*p* < .01
Average total # of medications	2.54 (1.53)	2.56 (1.54)	2.50 (1.54)	t(231) = 0.26	*P* > .05
# of antidepressants	0.25 (0.44)	0.22 (0.42)	0.33 (0.48)	t(231) = 1.77	*p* = .08
# of antipsychotics	1.33 (0.60)	1.37 (0.58)	1.26 (0.64)	t(231) = 1.23	*P* > .05
# of mood stabilizers	0.35 (0.58)	0.35 (0.58)	0.33 (0.54)	t(231) = 0.24	*P* > .05
# of bedtime sedatives	0.24 (0.47)	0.23 (0.46)	0.24 (0.49)	t(231) = 0.13	*P* > .05
# of antianxiety	0.17 (0.56)	0.17 (0.56)	0.18 (0.58)	t(231) = 0.17	*P* > .05
PANSS total	91.49 (19.31)	90.92 (19.14)	92.97 (19.8)	t(231) = 0.73	*P* > .05
PANSS positive	22.34 (6.19)	22.59 (6.41)	21.70 (5.60)	t(231) = 1.00	*P* > .05
PANSS negative	26.27 (7.37)	25.99 (7.24)	26.70 (7.69)	t(231) = 0.91	*P* > .05
PANSS general	42.92 (10.20)	42.34 (10.06)	44.40 (10.49)	t(231) = 1.38	*P* > .05
MoCA total	22.15 (4.57)	22.38 (4.51)	20.58 (4.71)	t(231) = 1.20	*P* > .05
Memory	2.37 (1.69)	2.42 (1.66)	2.26 (1.67)	t(231) = 0.66	*P* > .05
Visualspatial	3.06 (0.97)	3.10 (0.94)	2.97 (1.05)	t(231) = 0.93	*P* > .05
Executive	2.18 (1.28)	2.19 (1.26)	2.18 (1.32)	t(231) = 0.02	*P* > .05
Attention	4.66 (1.44)	4.74 (1.45)	4.45 (1.42)	t(231) = 1.35	*P* > .05
Language	4.15 (0.91)	4.15 (0.90)	4.15 (0.95)	t(231) = 0.01	*P* > .05
Orientation	4.78 (1.25)	4.75 (1.25)	4.83 (1.26)	t(231) = 0.43	*P* > .05

* Significance is at the 0.05 level (2-tailed).

** Significance is at the 0.01 level (2-tailed).

The average age of the smoker group was younger than that of the non-smoker group (35 vs. 42 years). An independent samples t-test revealed that the difference was statistically significant, t (231) = 4.31, *p* < .01.

The average years of education of smoker group was less than that of the non-smoker group (11 vs. 12 years). An independent samples t-test revealed that the difference was statistically significant, t (231) = 1.96, *p* < .05.

Among male patients, 77% were smokers. While in female patients, about 63% were smokers. A Pearson chi-square test indicated that the prevalence rates differed significantly between the groups, χ^2^ (1, *N* = 233) = 5.30, *p* < .05.

### Smoking status, length of stay, illness duration, illness severity, and comorbidity of substance use disorder

As shown in Table 1, the average length of stay (LOS) was 53 days for smokers and 64 days for non-smokers. An independent samples t-test showed the difference reached significance, t (231) = 4.31, *p* < .01.

The information of illness duration was available for129 of 233 patients. The average illness duration for smokers was shorter than that of non-smokers, 11 vs. 17 years. An independent samples t-test revealed that the difference was statistically significant, t (127) = 3.48, *p* < .01. This might be due to the fact that the smoker group was younger than the non-smoker group. A one-way ANOVA with age as the covariate showed that the difference of illness duration between the two groups remained significant, F(2, 126) = 72.79, *p* < .001.

Comparison of CGI scores for the two groups at admission showed no significant difference between smokers and non-smokers. Therefore, the overall illness severity was similar for the two groups.

In the smoker group, 64% of patients also had a substance/alcohol use disorder (SUD) comorbid diagnosis. In the non-smoker group, 17% of patients had a SUD comorbid diagnosis. A Pearson Chi-square test of independence showed the SUD rate in the smoker group was significantly higher than that in the non-smoker group, χ^2^(1, *N* = 233) = 42.53, *p* < .01.

### Smoking status and medication (especially antipsychotics) use

As shown in Table 1, comparisons of the total number of medications used, as well as the numbers of several major medication categories, i.e., antipsychotics, mood stabilizers, sedatives, antianxiety, and antidepressants, used in the two groups were conducted. None of the t-tests reached statistical significance.

Moreover, Chi-square tests were performed to examine the relationships between smoking status and the use of Clozapine, typical antipsychotics, and atypical antipsychotics (excluding Clozapine). The results showed there was no significant connections between smoking status and the use of Clozapine, χ^2^ (1, *N* = 233) = 0.56, *p* = .454, typical antipsychotics, χ^2^ (1, *N* = 233) = 0.423, *p* = .516, and atypical antipsychotics, χ^2^ (1, *N* = 233) = 0.333, *p* = .564.

Overall, these results indicate that smoking status is not significantly related to the numbers or types of antipsychotic medications used by patients with schizophrenia.

### Smoking status and psychiatric symptoms measured by the PANSS

Independent samples t-tests were conducted to compare the total and subscale scores of the PANSS of the two groups. As shown in Table 1, the two groups did not show significant difference in any of the t-tests.

Furthermore, fourteen independent samples t-tests were conducted to compare the scores of each individual item of the positive and negative subscales of the two groups. None of the t-test results reached statistical significance.

These results suggest that smoking status does not relate to patients’ psychiatric symptoms, including positive, negative, and general symptoms.

### Smoking status and cognitive functions measured by the MoCA

For smokers, 122 out of 165 patients had a MoCA score of less than 26, indicating that 74% of smokers showed mild cognitive impairment (MCI). For non-smokers, 49 out of 66 patients had a MoCA score of less than 26, suggesting that 74% of non-smokers demonstrated MCI. Therefore, the same percentage of patients with MCI presented in both the groups.

Furthermore, independent samples t-tests were conducted to compare the total and subdomain scores of the MoCA of the two groups. As shown in Table 1, none of the t-tests reached statistical significance.

Thus, overall there was no significant association between smoking status and patients’ general cognitive function and the cognitive subdomains measured by the MoCA.

## Discussion

The smoking prevalence rate was 72% for patients with schizophrenia or schizoaffective disorders, which was higher than the rate for patients with bipolar disorder (67%), MDD (40%), and other psychiatric disorders (58%). According to a most recent (2015–2021) Statistics Canada report on smokers by age group, the average smoking rate was about 18 −19% for Canadians age 18 to 64 years^43^. Thus, the smoking prevalence in this patient group is almost four times that of the general population.

The high smoking prevalence rate found among patients with schizophrenia in this study further confirms numerous previous findings in the literature. Moreover, our results are consistent with the converging observation that the smoking prevalence in patients with schizophrenia is also higher than patients with other psychiatric disorders^2,10,27^.

### Smoking status and demographic characteristics in patients with schizophrenia

Our results show that male patients are more likely to smoke than female patients. This finding is confirmed by the literature, other than a few studies that find smoking status to be independent of gender^5,13,14,18,21,22,25,30^. The average age of the smoker group was younger than that of the non-smoker group. This is in line with many previous findings^3,13,21,28,29^. However, several studies did not find significant age difference between smoker and non-smoker patients^5,19,30^. The average years of education for the smoker group was less than that of the non-smoker group in our study, which confirms the findings of numerous other studies^3,17,18^. Again, there are also studies that find no difference in education level between the two groups^19,30^.

In the existing literature investigating smoking and schizophrenia, a wide variety of patient groups and clinical settings were involved. The demographic characteristics of patient groups can be very diverse. For example, the average age of a treatment-resistant schizophrenia patient group could be much older than that of a first-episode psychosis schizophrenia patient group. This might have contributed to the fact that the relationships found between smoking status and demographic characteristics are highly mixed. Nevertheless, consistent with many previous findings with different patient groups, patients who smoke and needed TMH care (those showing more acute and severe psychotic symptoms), seem to be associated with younger age, male gender, and less education.

### Smoking status, illness duration, SUD comorbidity, and medication use

This study found illness duration for the smoker group to be significantly shorter than that for the non-smoker group, which is compatible with findings from several previous studies^13,21^. Nevertheless, other studies have found that smoking status is not related to illness durations^5,19,30^.

The proportion of patients who had SUD comorbidity was significantly higher in the smoker group than in the non-smoker group. This supports previous findings that smoking patients were more likely to have a secondary SUD diagnosis [or alcohol or cannabis dependence] than non-smoker patients^2,3,5,8,13^. However, other studies have shown that smoking was independent of alcohol or other substance use^17,22^.

Finally, our results showed that medication use, including antipsychotic use, was independent of smoking status. This is consistent with the findings of Iasevoli et al. but conflicts with findings from other studies that smoking is associated with first generation antipsychotics use, conventional [atypical] antipsychotics use, and a few other drugs including clozapine administration^5,17,19,22^.

### Smoking status, illness severity, and psychiatric symptoms

Our results showed that the overall illness severity measured by the CGI, as well as the positive, negative, and general psychiatric symptoms assessed by the PANSS, were similar for the two groups. This is consistent with many previous findings that smoking status was not associated with psychiatric symptoms measured by PANSS, SAPS, or SANS^2,3,18,20,25,26^. However, there are also reports indicating that smoking has significant effects - whether beneficial or adverse - on symptoms^17,19,21^.

The most accepted self-medication hypothesis suggests that patients use smoking to self-medicate their symptoms and the side effects of antipsychotic medicines. Though testing the hypothesis is not the purpose of this study, the current results are not in favor of this theory, since patients in the smoker group did not show alleviated symptoms compared to non-smokers.

### Smoking status and cognitive function

In the current study, patients in the smoker and non-smoker groups did not show any difference in the MoCA total and subdomain scores. Furthermore, the same percentage of patients were classified as having at least mild cognitive impairment measured by the MoCA, regardless of whether they were smokers or non-smokers. Our results confirm previous findings that smoking status did not relate to cognitive functions^13,23,31^. However, some research showed that smoking status has a significant effect on cognitive functions, with the effect being either positive or negative^3,6,18,19,25,28,29^. Again, our findings of similar cognitive function in the two groups seem to cast doubt on the self-medication hypothesis.

Most previous studies have involved a large variety of cognitive tasks that measured different specific cognitive domains. In the current study, patients’ cognitive function was evaluated by the MoCA, which includes six cognitive subdomains. The MoCA has been proven to be an effective and valid cognitive assessment tool for fast evaluation of global cognitive impairment in patients with schizophrenia^44–46^. Thus, the current findings provide a brief yet more comprehensive view of the relationship between smoking status and overall cognitive function.

## Limitations and clinical implications

This study aimed to investigate smoking status in acute inpatients who needed tertiary mental health services, specifically focusing on patients who could not be treated in general hospitals psychiatry wards due to their acute and severe psychotic symptoms. The smoking prevalence, connections between smoking status and demographic, clinical, and cognitive factors were investigated within one sample. Potential confounding effects due to different patient populations were minimized. However, there are some limitations that come with the retrospective chart review nature of this study. For example, some information regarding smoking such as number of cigarettes consumed daily were either not recorded in the charts or difficult to capture accurately. Therefore, we were only able to consider the smoking status as a binary variable and were unable to conduct multivariate analysis. In addition, though we were able to examine the connections between smoking status and several demographical, clinical, and cognitive variables, no causal relationship could be determined.

Substance use comorbidity is quite common among patients with a mental illness. In Canada, at least 20% of people with a mental illness have a co-occurring substance use disorder^46^. For individuals with schizophrenia, this percentage may be up to 50%^47^. In the current study, 55% of the patients had concurrent SUD. For the smoker patients, the rate was as high as 64%. A study investigating SUD in people with schizophrenia across Europe reported lower comorbid rates than those reported from USA and Canada. The lifetime SUD comorbid rates was 35% in UK, 21% in Germany, and 19% in France^48^. Regarding smoking and SUD, it appears Europe is facing less challenges.

Given the higher smoking prevalence among patients with severe schizophrenia and the increased comorbidity of substance use disorders (SUD) in smokers, it is crucial for facilities and hospitals that accept and treat these patients to be aware of the associated challenges, risks, and complexities. Our study also found no link between smoking and less severe psychotic symptoms, underscoring the importance of encouraging smoking cessation. Moreover, a recent study reported patients with psychotic disorders who smoke cigarette had a significantly reduced life expectancy than non-smokers with the disorders^49^. The risks were equivalent to those associated with a diagnosis of comorbid alcohol or opioid use disorder. At the individual level, treatment plans and behavioral interventions should address each patient’s smoking and substance use status. Promoting and supporting smoking and substance use cessation is essential, and plans should be in place to manage withdrawal symptoms. At the organizational level, enforcing a non-smoking policy and prohibiting the consumption of illicit drugs are vital to maintaining a supportive treatment environment.

## Conclusion

Taken together, this study found that the smoking prevalence rate is remarkably high (72%) among inpatients with schizophrenia who present with acute and severe psychotic symptoms. It is greater than that in patients with other acute psychiatric disorders. Smoking appeared to be associated with male gender, less education, younger age, shorter illness duration, higher rate of SUD comorbidity, and less length of stay in hospital. Smoking did not relate to patients’ illness severity, medication use, psychiatric symptoms, or cognitive functions.

## Data Availability

The data underlying this study cannot be shared publicly due to ethical considerations. Data will be shared on reasonable request to the corresponding author with permission of Interior Health Authority.

## Abbreviations

ANOVA: Analysis Of Variance
CGI: Clinical Global Impression scale
LOS: Length of Stay
MCI: Mild Cognitive Impairment
MoCA: the Montreal Cognitive Assessment
NRT: Nicotine Replacement Therapy
PANSS: Positive and Negative Symptom Scale
SPSS: Statistical Package for the Social Sciences
SUD: Substance Use Disorder
TMH: Tertiary Mental Health
